# A Pilot Study About the Dysfunction of Adipose Tissue in Male, Sleep Apneic Patients in Relation to Psychological Symptoms

**DOI:** 10.3389/fpsyt.2019.00527

**Published:** 2019-07-25

**Authors:** Georgia Trakada, Pantelis T. Nikolaidis, Nicholas-Tiberio Economou, Dimitrios Sakkas, Giorgos Giagkou, Stratigoula Sakellariou, Konstantina Kyriakopoulou, Efstratios Patsouris, Luigi Ferini-Strambi, Lemonia Velentza, Anastasios Kallianos, Thomas Rosemann, Beat Knechtle, Asimina Mitrakou

**Affiliations:** ^1^Department of Clinical Therapeutics, National and Kapodistrian University of Athens School of Medicine, Alexandra Hospital, Athens, Greece; ^2^Exercise Physiology Laboratory, Nikaia, Greece; ^3^1st Department of Pathology, National and Kapodistrian University of Athens School of Medicine, Athens, Greece; ^4^Radiology (Ultrasound) Department, Athens Medical Center, Athens, Greece; ^5^Department of Clinical Neurosciences, Neurology - Sleep Disorders Center, IRCCS San Raffaele Scientific Institute, Milan, Italy; ^6^Institute of Primary Care, University of Zurich, Zurich, Switzerland

**Keywords:** obstructive sleep apnea, Hospital Anxiety and Depression Scale, adipose tissue, abdominal fat, biopsy

## Abstract

**Introduction:** Obstructive sleep apnea (OSA) and its cardiometabolic alterations are closely associated with visceral obesity. Patients with OSA frequently present with symptoms of depression and anxiety. Although these subjective symptoms of OSA are the result of complex biological dysregulation, it remains unclear if they have a direct effect on the dysfunction of adipose tissue.

**Methods:** In a pilot, prospective, randomized study, we evaluated 10 recently diagnosed male patients with severe OSA by full polysomnography (PSG) and 4 male non-apneic subjects matched for age and body mass index (BMI) with abdomen adipose tissue biopsies. Subjects with diabetes/prediabetes and cardiovascular and psychiatric diseases and who are current smokers were excluded. All patients underwent anthropometric measurements and completed the following questionnaires: Epworth Sleepiness Scale (ESS), Fatigue Severity Scale (FSS), and Hospital Anxiety and Depression Scale (HADS-A and HADS-D). Fasting venous blood samples were collected on the day after PSG, between 8:00 and 9:00 a.m., after an overnight fast. Fat biopsies were performed at the same time periods and adipose tissue samples of 300 mg were obtained from abdominal fat. Fat cell size, extent of fibrosis, vascularity, leukocyte common antigen inflammatory infiltration, and tissue macrophage accumulation were microscopically evaluated.

**Results:** The mean age of the group was 47.4 ± 13.8 years, with mean BMI of 35.8 ± 4.8 kg/m^2^ and mean apnea-hypopnea index of 79.4 ± 46.1 events per hour of sleep (severe OSA). HADS-A and HADS-D scores were 5.8 ± 2.3 (3.0–8.0) and 4.7 ± 2.3 (2.0–8.0), respectively. HADS-A score correlated positively with macrophage accumulation in fat biopsy (r = 0.82, p = 0.047), whereas ESS, FSS, and HADS-D did not. Severity of fibrosis correlated largely with waist circumference (r = -0.66, p = 0.038) and neck circumference (r = -0.790, p = 0.006). Respiratory events correlated negatively with the extent of vascularization of adipose tissue (r = -0.614, p = 0.05).

**Conclusions:** In the preliminary results of our pilot study, we assessed that the symptoms of anxiety mainly contribute to macrophage accumulation, whereas the increased number of respiratory events reduces the extent of vascularization in visceral fat in OSA. Based on this observation, further larger studies are required to verify if anxious OSA patients are more vulnerable to the metabolic manifestations of the syndrome.

## Introduction

Obstructive sleep apnea (OSA) is a chronic condition characterized by repetitive collapse of the upper airway during sleep leading to intermittent hypoxia (IH) and recurrent arousals from sleep. OSA is a prevalent disorder particularly among middle-aged, obese men, although its existence in women as well as in lean individuals is increasingly recognized (1). OSA is associated with considerable morbidity and mortality, and numerous studies indicate a causal relationship between OSA and hypertension, cardiovascular disease, insulin resistance (IR), and diabetes mellitus (2).

Affective disorders such as depression and anxiety are commonly reported in patients with OSA. Previous studies hypothesized that OSA, psychological symptoms, and metabolic disease are the diverse consequences of underlying biological, metabolic, and neurologic dysregulation through low-grade inflammation, oxidative and nitrosative stress, and neurotransmitter imbalances (3–5). Each individual can express multiple disease states according to the underlying biological dysregulation (4).

For many years, adipose tissue was regarded only as an energy storage place. Now, visceral adipose tissue (VAT) is considered as a high, metabolically active tissue and a local source of pro-inflammatory factors, closely associated with metabolic risk (6). Visceral obesity is associated with OSA. Apnea-hypopnea index (AHI) more often correlates with intra-abdominal fat than with subcutaneous fat in the neck region or parapharyngeal fat (7). Also, waist circumference is a better predictor of OSA than body mass index (BMI) (8). Stress-induced, glucocorticoid-mediated visceral obesity and metabolic syndrome manifestations are common in chronically stressed individuals and psychiatric populations due to a blunted circadian rhythm of cortisol secretion and mild hypercortisolism (9, 10).

The aim of our study was to evaluate the complex interactions among OSA, psychological symptoms, and abdominal adipose tissue dysfunction.

## Patients and Methods

### Study Population

Ten male patients with newly diagnosed, severe OSA and four non-apneic male subjects, matched for age and BMI (controls), were selected for the present study from our outpatient clinic in the Department of Clinical Therapeutics, National and Kapodistrian University of Athens School of Medicine, Alexandra University Hospital of Athens. Written informed consent was obtained from all individual participants included in the study and the study protocol was approved by the Ethical Committee of the Alexandra University Hospital before the initiation of the study (approval no. 301/30.07.2013). The recruitment and assessment of the patients were performed by one pulmonologist (GT).

OSA was diagnosed as an AHI of ≥5 events per hour of sleep with associated symptoms or comorbidities or an AHI of ≥15, regardless of associated symptoms or comorbidities, according to the American Academy of Sleep Medicine diagnostic criteria (11).

Exclusion criteria were known diabetes mellitus or fasting glycemia [fasting glucose ≥126 mg/dl or glycosylated hemoglobin (HbA1c) > 6.5%], current smoking, cardiovascular or cerebrovascular disease, uncontrolled hypertension or other chronic disease, known psychiatric disease, systemic medication use, and previous diagnosis and/or treatment for OSA and/or other sleep disorders.

At baseline, each patient was evaluated by medical history and clinical examination, underwent spirometry, and completed a detailed questionnaire about sleep habits.

### Sleep Questionnaires

All patients answered the Epworth Sleepiness Scale (ESS), the Fatigue Severity Scale (FSS), and the Hospital Anxiety and Depression Scale (HADS). ESS is a self-report questionnaire that evaluates subjective sleep propensity, and a score of ≥10 indicates excessive sleepiness (12). FSS is a method of evaluating the impact of fatigue in daytime functioning, and a score of ≥4 is interpreted as abnormal, especially in subjects who suffer from sleep disorders (13). Finally, HADS is a reliable instrument for detecting and separating the states of depression and anxiety, by excluding somatic symptoms, in patients attending a general medical clinic (14). Scores of >10 are indicative of psychological morbidity, whereas scores between 8 and 10 are considered borderline.

### Sleep Study

A standard full-night polysomnography (PSG) was performed on each patient on the same night of the initial evaluation (Alice, Respironics, Murrysville, PA). Sleep records were scored according to standard criteria and manually revised by an expert (15). Apnea was defined as a drop in the peak signal excursion by ≥90% of the pre-event baseline using an oronasal thermal sensor (diagnostic study), a positive airway pressure device flow (titration study), or an alternative apnea sensor when the duration of ≥90% drop in the sensor signal was ≥10 s. Hypopnea was defined as the breathing reduction of ≥30% of the pre-event baseline that exceeded 10 s and was associated with ≥3% oxygen desaturation from the pre-event baseline or with an arousal. Arousal was defined as an abrupt shift in electroencephalograph frequency, including alpha, theta, and/or frequencies greater than 16 Hz (but not spindles) that last at least 3 s, with at least 10 s of stable sleep preceding the change and increased mylohyoid electromyographic signal of >1 s during rapid eye movement (REM) sleep. AHI was defined as the total number of apneas and hypopneas per hour of electroencephalographic sleep.

### Fat Biopsy

Fasting venous blood samples were collected on the day after PSG, between 8:00 and 9:00 a.m., after an overnight fast, for the measurement of glucose, HbA1c, lipid, and liver profile. Body composition was analyzed with a bioelectrical impedance system (Siemens Acuson S 2000, Germany). Fatty liver was assessed by ultrasonography (Tanita bioimpedence TBF-300 body composition analyzer).

Fat biopsies were also performed to obtain adipose tissue samples (300 mg) by needle biopsy from the abdomen without anesthesia, as described previously (16). One block of formalin-fixed paraffin-embedded tissue was prepared in each case and 4-μm-thick sections were processed. Adipose tissue was microscopically examined by an experienced pathologist (SS), unaware of the clinical information, to evaluate the fat cell size, extent of fibrosis, vascularity, leukocyte common antigen (LCA) inflammatory infiltration, and tissue macrophage accumulation. The size of fat cells was estimated on eosin-hematoxylin standard stained sections. Masson trichrome histochemical staining was performed for the assessment of fibrosis. Immunohistochemistry against CD34, LCA, and CD68 antibodies was conducted to depict vascular structures, B and T lymphocytes, and tissue macrophages, respectively. Severity of fibrosis and density of vascular structures, inflammatory cells, and tissue macrophages were semi-quantitatively assessed on a scale from 0 to 6, where 0 corresponded to absence and 6 to the highest degree of the examined feature. Immunohistochemical analysis was performed using the EnVision Staining System (Dako, Glostrup, Denmark) with 3,3′-diaminobenzidine as a chromogen and a PT Link, Pre-Treatment Module according to standard protocols. Primary antibody incubation was omitted as negative control. Samples known to strongly immunoreact to the specific antibodies were used as positive control.

Analysis was performed by SPSS for Windows. Normality was examined with the Kolmogorov-Smirnoff test. Descriptive results for continuous variables were expressed as mean ± SD. Univariate and multivariate analyses were used to determine possible correlations between anthropometric data, laboratory findings, and questionnaires’ scores and indices of apnea (AHI and SaO_2_). The OSA and the control groups were compared for severity of fibrosis, density of vascular structures, inflammatory cells, and tissue macrophages using an independent t test, and Cohen’s d was used to evaluate the magnitude of any difference. Statistical significance was defined at a level of 5% (P ≤ 0.05).

## Results

### Baseline Characteristics of the Study Population

**Figure 1:**
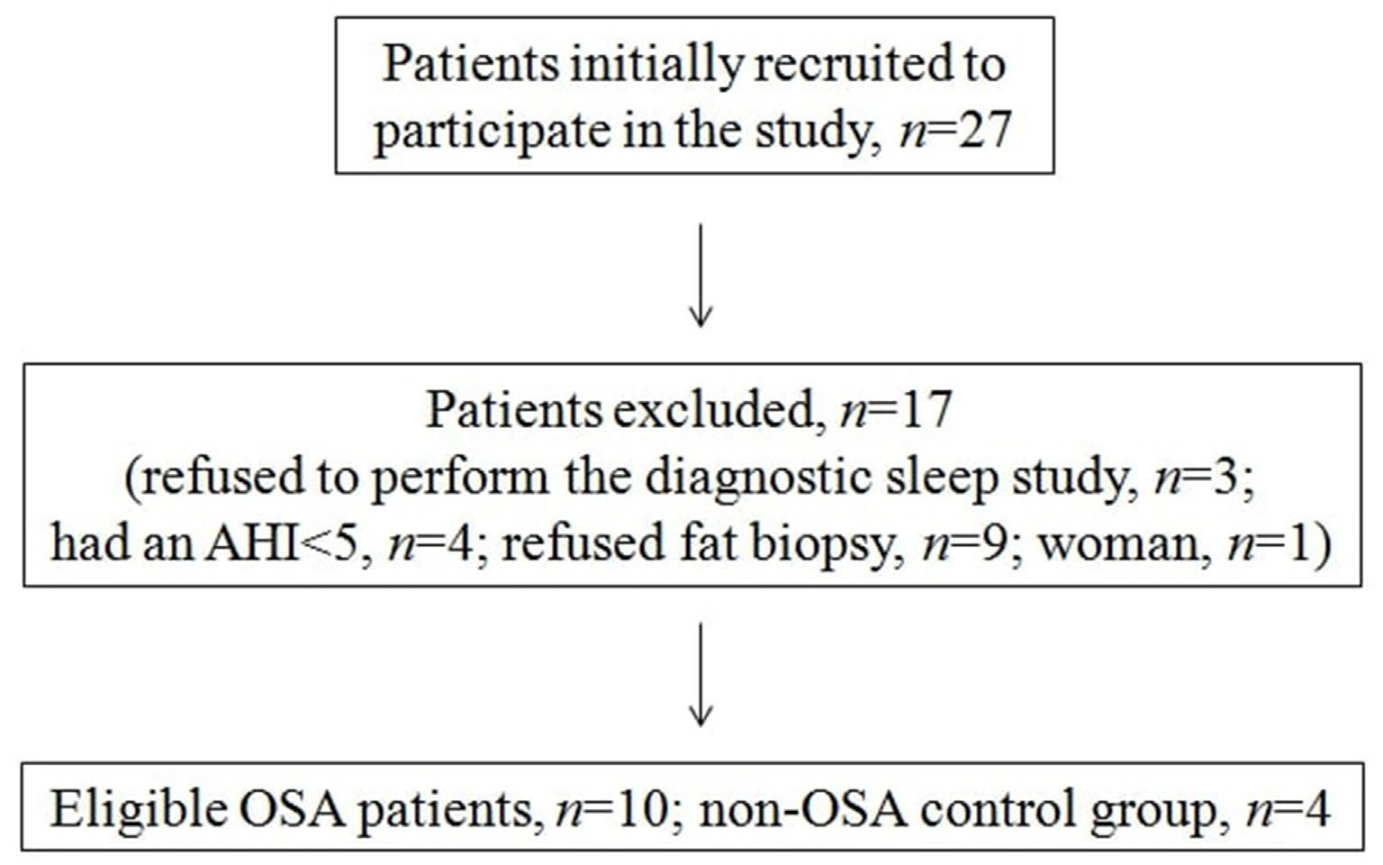
Study protocol presenting the number of patients recruited, excluded, and included.

A total of 27 patients were recruited during our study period. Three patients refused to perform the diagnostic sleep study, four patients had an AHI of <5, and nine patients who refused fat biopsy were excluded from the study. Furthermore, women were excluded (n = 1) resulting in a final sample of 10 newly diagnosed OSA patients. A total of 4 non-apneic male subjects, matched for age and BMI, served as controls. The patients’ dispositions according to the study protocol are presented in **Figure 1**. The baseline characteristics of the study population are presented in **Table 1**. No difference was observed in any of these characteristics between the OSA and the control groups, except for forced expiratory volume in 1 s (FEV_1_)/forced vital capacity (FVC; lower score in the OSA group).

**Table 1:** Baseline characteristics of the OSA patients and control group (mean ± SD).

Parameter	OSA group	Control group
Age (years)	47.4 ± 13.8	51.3 ± 11.3
BMI (kg/m^2^)	35.8 ± 4.8	34.2 ± 4.1
Neck circumference (cm)	44.3 ± 3.2	45.3 ± 2.3
Waist circumference (cm)	126.6 ± 16.5	133.8 ± 17.9
Hip circumference (cm)	109.8 ± 32.4	126.3 ± 2.1
Systolic arterial blood pressure (mmHg)	132.1 ± 8.1	133.8 ± 5.0
Diastolic arterial blood pressure (mmHg)	90.4 ± 6.2	90.0 ± 5.0
FVC (%)	92.0 ± 13.9	94.8 ± 6.7
FEV_1_ (%)	95.7 ± 13.9	90.1 ± 11.3
FEV_1_/FVC	84.6 ± 4.1	94.8 ± 5.4*
SaO_2_	96.4 ± 1.3	N/A
Glucose (mg/dl)	96.3 ± 8.6	103.5 ± 3.2
HbA1c	5.7 ± 0.5	N/A
Cholesterol	216.5 ± 42.8	206.8 ± 40.4
Triglycerides	166.0 ± 91.5	129.0 ± 21.2
HDL	36.5 ± 14.1	36.8 ± 7.0
LDL	145.6 ± 24.5	150.5 ± 39.7
AST	26.9 ± 9.1	28.3 ± 4.2
ALT	35.9 ± 24.3	41.8 ± 20.2
LDH	238.0 ± 47.2	207.5 ± 7.6

ALT, alanine aminotransferase; AST, aspartate aminotransferase; LDH, lactate dehydrogenase; LDL, low-density lipoprotein; HDL, high-density lipoprotein. *p = 0.001.

ESS score was 13.0 ± 5.3 (6.0–24.0) and 14.5 ± 4.7 (10.0–21.0), FSS score was 3.6 ± 1.5 (1.6–5.6) and 3.4 ± 1.8 (1.5–5.6), HADS-A score was 5.8 ± 2.3 (3.0–8.0) and 5.5 ± 2.1 (3.0–8.0), and HADS-D score was 4.7 ± 2.3 (2.0–8.0) and 5.3 ± 2.8 (2.0–8.0) in the OSA and the control groups, respectively. All patients suffered from severe OSA (AHI 33.7–189.4 events per hour of sleep). The sleep data of OSA patients are presented in **Table 2**. As expected, the AHI of the OSA group was higher (79.4 ± 46.2 versus 2.4 ± 1.0 events per hour of sleep, respectively, p = 0.007) and the minSaO_2_ was lower than in the control group (78.7 ± 8.4% versus 88.3 ± 1.7%, respectively, p = 0.047), whereas no difference was observed in the mean SaO_2_ (89.8 ± 4.6% versus 91.4 ± 0.9%, respectively, p = 0.500).

**Table 2:** Sleep characteristics of the study population (mean ± SD).

Total recording time (min)	233.9 ± 116.0
TST (min)	204.4 ± 114.7
SE (%)	85.8 ± 13.4
Sleep onset (min)	12.4 ± 14.0
N1 (min)	35.8 ± 61.8
N2 (min)	108.5 ± 43.2
N3 (min)	49.5 ± 68.2
REM (min)	10.3 ± 16.0
Apneas+hypopneas (n)	234.4 ± 129.8
Obstructive apneas (n)	89.9 ± 66.4
Central apneas (n)	23.4 ± 28.5
Mixed apneas (n)	15.2 ± 32.4
Hypopneas (n)	95.2 ± 76.2
AHI	79.4 ± 46.1
Mean SaO_2_ (%)	90.0 ± 4.6
Min SaO_2_ (%)	78.7 ± 8.4
SaO_2_ < 90% (%)	10.3 ± 21.8

N1, stage 1; N2, stage 2; N3, stage 3–4; TST, total sleep time.

### Fat Biopsy Results

The body composition data of the OSA group, according to the impedance analysis, are as follows: body fat (BF) mass (BFM) 40.6 ± 13.4 kg and BF 34.4 ± 6.9%, free fat mass (FFM) 74.7 ± 8.0 kg, total body water 54.7 ± 5.9%, and basal metabolic rate 2281 ± 302 kcal/day. Adipose tissue examination, as assessed on a scale from 0 to 6, revealed 3.8 ± 1.0 severity of fibrosis, 2.5 ± 1.4 density of vascular structures, 1.4 ± 1.0 B and T lymphocytes, and 3.2 ± 2.1 tissue macrophages. The data of each patient separately are presented in **Table 3**.

**Table 3:** Data of fat biopsy and body composition.

Patient (n)	Severity of fibrosis (0–6)	Density of vascular structures (0–6)	B and T lymphocytes (0–6)	Tissue macrophages (0–6)	Fat mass (kg)	FFM (kg)
1	5	1	1	2	26.1	65.9
2	2	4	1	6	61.2	70.2
3	3	4	2	6	43.9	79.1
4	4	1	1	3	43.2	90.6
5	4	1	1	5	29.2	69.3
6	3	2	1	1	77.9	93
7	3	4	1	2	50	70
8	4	4	1	1		
9	5	2	4	5	24.4	63.3

Waist circumference correlated positively with BFM (r = 0.971, p < 0.001) and BF (r = 0.852, p = 0.004). Also, neck circumference correlated with BFM (r = 0.880, p = 0.002) and BF (r = 0.732, p = 0.025). Hip circumference correlated with neither BFM (r = -0.181, p = 0.641) nor BF (r = -0.416, p = 0.265). Sleep efficiency (SE) correlated negatively with waist circumference (r = -0.791, p = 0.006), neck circumference (r = -0.790, p = 0.006), BFM (r = -0.787, p = 0.012), and BF (r = -0.767, p = 0.016).

**Figure 2:**
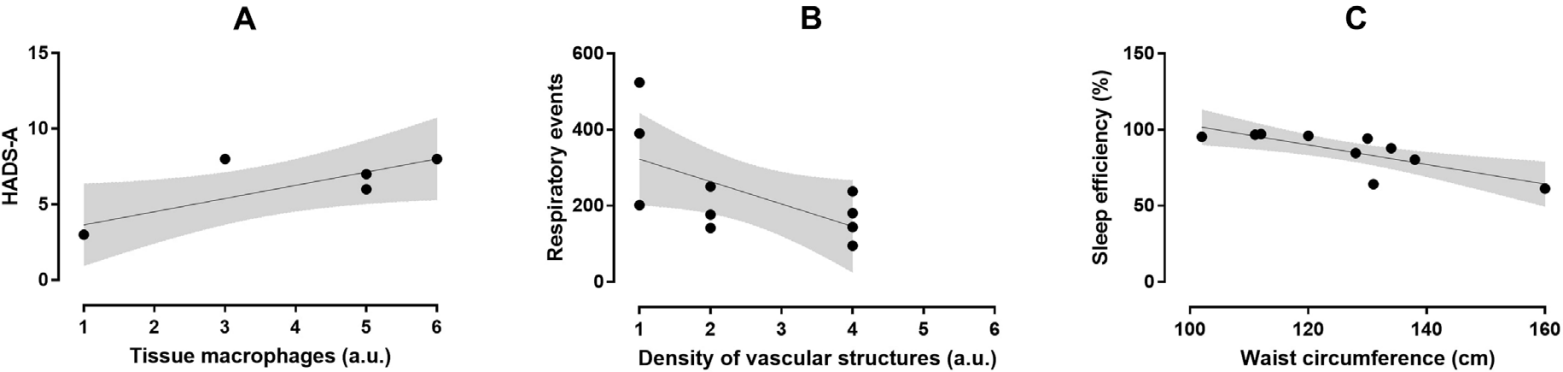
Relationship between macrophage accumulation in fat biopsy and HADS-A score (r = 0.82, p = 0.047; **A**), density of vascular structures and respiratory events (r = -0.614, p = 0.05; **B**), and SE and waist circumference (r = -0.791, p = 0.006; **C**) in the OSA group.

**Figure 3:**
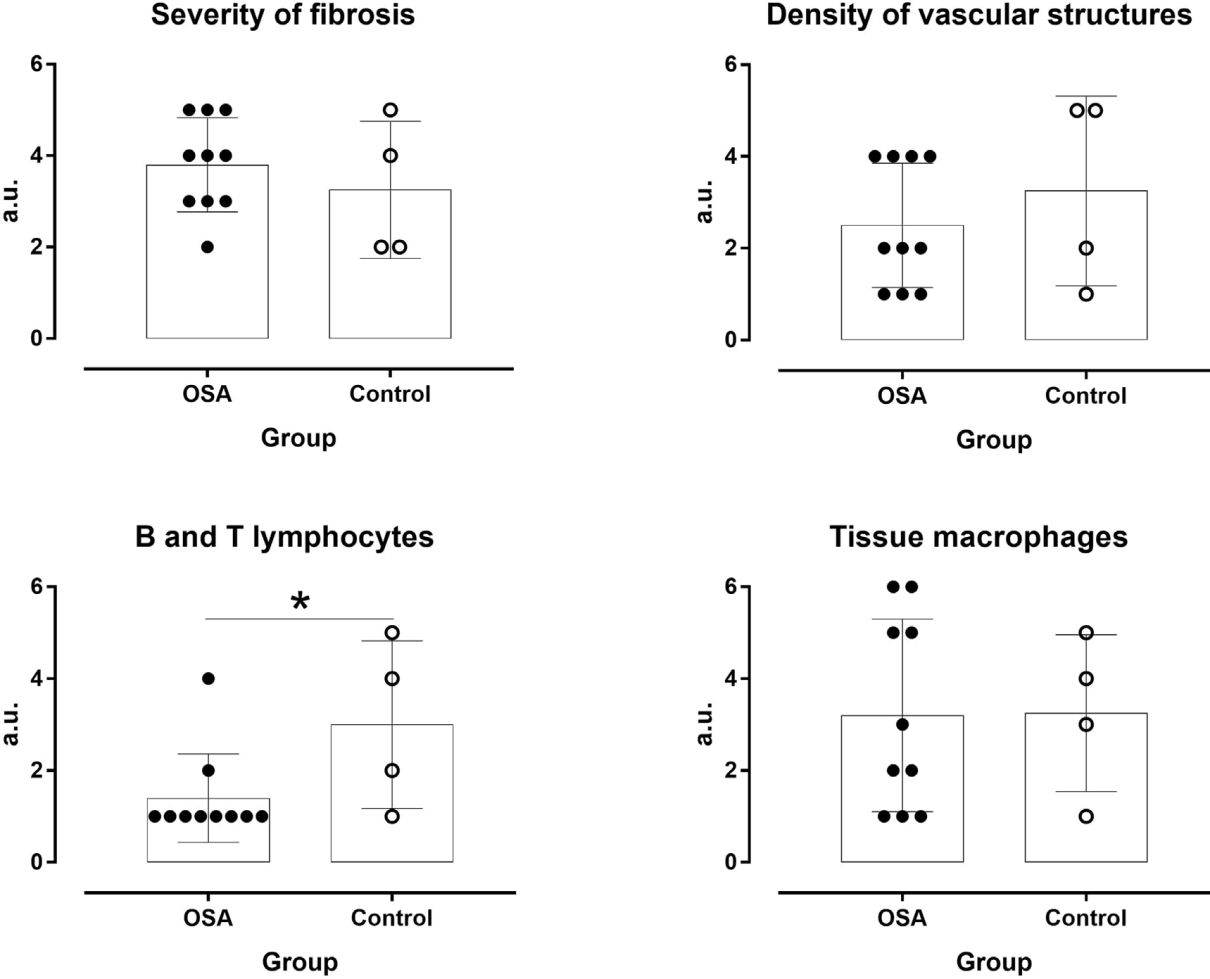
Severity of fibrosis and density of vascular structures, inflammatory cells, and tissue macrophages in OSA patients and controls. *P ≤ 0.05.

In the OSA group, macrophage accumulation in fat biopsy correlated positively with HADS-A score (r = 0.82, p = 0.047) but not with ESS, FSS, and HADS-D scores (**Figure 2**). Severity of fibrosis correlated largely with waist circumference (r = -0.56, p = 0.094), whereas density of vascular structures correlated positively with subjective duration of sleep (r = 0.68, p = 0.062) and negatively with respiratory events (r = -0.614, p = 0.05). The OSA group had lower B and T lymphocyte inflammation scores than the control group (p = 0.050, d = -1.09, large ES), whereas no difference was observed in severity of fibrosis (p = 0.441, d = 0.43, small ES), density of vascular structure (p = 0.433, d = -0.43, small ES), and tissue macrophages (p = 0.967, d = -0.03, trivial ES; **Figure 3**). In the control group, severity of fibrosis correlated with HADS-D (r = -0.99, p = 0.011) and inflammation with FSS (r = -0.99, p = 0.010), whereas no other correlation was shown.

## Discussion

To our knowledge, this is the first study to use fat biopsy to assess a complex interaction among OSA, psychological symptoms, and dysfunction of VAT. According to our findings, the dysregulation of the abdomen fat in OSA is triggered by different stimuli. Reduced SE is associated with increased waist circumference and fat mass, whereas anxiety symptoms promote macrophage accumulation, and repetitive respiratory events during sleep—apneas and hypopneas—reduce the extent of vascularization in abdomen fat.

In accordance to previous studies, the waist circumference of our population correlated with fat mass and BF (17). The measurement of abdominal obesity is known to be strongly associated with increased cardiometabolic risk, cardiovascular events, and mortality (17). Although waist circumference is a crude measurement, it correlates with obesity and visceral fat amount and is a surrogate marker for IR (17). Moreover, waist circumference predicts better OSA than neck or hip circumference (8). Visceral obesity is characterized by an overgrowth of adipose tissue that leads to the formation of hypoxic areas within this tissue due to mechanical reasons. Adipose tissue hypoxia is one of the earliest events in adipose tissue dysfunction that induces a local state of ﬁbrosis in the tissue (18).

The novel finding of our study was the relation between anxiety symptoms in OSA patients and the accumulation of macrophages in adipose tissue, which represents the key event in the induction of inflammation. Interestingly, depressive symptoms did not correlate with the accumulation of macrophages. According to current evidence, macrophages progressively infiltrate the adipose tissue during obesity, initiate the pro-inflammatory response by producing tumor necrosis factor-α (TNF-α) and interleukin (IL)-6, and block adipocyte insulin action, a contributing factor in the development of IR and type 2 diabetes mellitus (19, 20). The overgrowth of adipose tissue leads to local tissue hypoxia, fibrosis, and finally adipocyte death by necrosis, which promotes the further recruitment of macrophages to scavenge the resulting cell debris (21). However, what exactly triggers the initial activation and infiltration of macrophages during obesity remains unclear. According to our data, symptoms of anxiety could be implicated in the accumulation of macrophages in abdomen fat.

The prevalence of anxiety in OSA ranges between 11% and 70% (22). According to a recent study, OSA patients have 3.68 times increased odds of anxiety and 2.88 times increased odds of severe psychological distress (23). Patients with OSA and anxiety differ in brain imaging of metabolic and structural disorders from those with OSA alone (24). Increased sympathetic activity associated with anxiety, brain alterations, and inflammatory mediators can contribute to the pathogenesis of OSA and vice versa (10).

We also confirmed a negative effect of the number of apneas and hypopneas during sleep on the extent of vascularization into visceral fat. Cumulative evidence points a causal relationship between OSA and cardiovascular and metabolic dysfunction independent of obesity (10). Several factors, such as oxidative stress, inflammation, and hypersecretion of adipocyte-derived hormones due to repetitive, short periods of asphyxia and hypoxemia and sleep deprivation may contribute to the complicated pathophysiologic cascade of events in OSA. Especially, this particular form of IH observed in OSA, with repetitive short cycles of desaturation followed by rapid reoxygenation, seems to dysregulate glucose metabolism through various mechanisms. We have shown previously that, in non-diabetic male OSA patients without comorbidities, the number of respiratory cessation events, the indices of hypoxemia during sleep, and the arousal index were associated with increased fasting glucose and HbA1c levels (25). Furthermore, a U-share relation was observed between fasting plasma glucose levels and OSA severity, regardless of obesity, as expressed by BMI (26). Moreover, the variability of glucose levels and arterial pressure was also associated with the number of apneas and hypopneas and the degree of oxygenation during sleep (27). Finally, male OSA patients with no prior history of cardiovascular disease had significantly higher C-reactive protein levels and lower flow-mediated dilation values compared to control subjects, indicating the possible presence of subclinical atherosclerosis and subsequent increased risk for developing cardiovascular disease (28). The impact of respiratory events on visceral fat may represent another possible underlying mechanism of the cardiometabolic risk in OSA patients.

Both the state of hyperarousal due to anxiety symptoms and the sleep fragmentation due to repetitive apneas and hypopneas reduce SE. According to our data, reduced SE further increases fat mass and waist circumference. All these complex pathophysiologic mechanisms could interact in a vicious cycle that further aggravates sleep apnea.

Another interesting finding of our study was the observed difference in adipose tissue composition between apneic, obese male and non-apneic, obese controls. It is well known that macrophages act either as pro-inflammatory (M1 phenotype) to stimulate inflammation or as “meta-inflammatory” (M2 phenotype) to reduce inflammation (29). M2 adipose tissue macrophages are mainly maintained by the action of lymphocytes. However, obesity suppresses the recruitment of these lymphocytes to abdominal fat and down-regulates tolerogenic CD4^+^ T-regulatory cells, which could also lead to meta-inflammation (30, 31). As our OSA patients had lower B and T lymphocyte inflammation scores than the control group, we could hypothesize that sleep apnea further suppresses the “meta-inflammatory” process and increases the cardiometabolic risk.

The main strength of our study is the novelty of our data. However, the study has several limitations. One is the relatively restricted number of patients and the lack of women in the sample; certainly, our findings have to be verified on a larger scale. Moreover, due to the small number of participants, we cannot distinguish different groups according to severity of OSA (mild, moderate, or severe) or according to different phenotypes (sleepy versus non-sleepy, fatigued versus non-fatigued, etc.). OSA is a heterogeneous disease characterized by different clinical outcomes and prognosis despite similar AHI. Finally, we did not measure markers of inflammation, such as TNF-α, or Il-6, or indices of IR in the blood.

According to our data, anxiety symptoms may promote macrophage accumulation, and an increased number of respiratory events can reduce the extent of vascularization in visceral fat, whereas reduced SE can worsen indices of obesity in severe OSA. These complex interactions should be evaluated in larger studies to clarify the mechanisms underlying inflammation in OSA and to identify potential targets for clinical intervention.

## Author Contributions

GT and AM conceived the study and sought ethical approval. GT and AM completed the assessments. GT and PN analyzed the data. All authors contributed in writing the paper and approved the final version of the manuscript.

## Funding

The study was financially supported by a Grant from the Hellenic Thoracic Society.

## Conflict of Interest Statement

PN was employed by company Exercise Physiology Laboratory, Nikaia, Greece.

The remaining authors declare that the research was conducted in the absence of any commercial or financial relationships that could be construed as a potential conflict of interest.
